# Multivariate analysis of breast tissue using optical parameters extracted from a combined time-resolved fluorescence and diffuse reflectance system for tumor margin detection

**DOI:** 10.1117/1.JBO.28.8.085001

**Published:** 2023-08-23

**Authors:** Erica Dao, Gabriella Gohla, Phillip Williams, Peter Lovrics, Fares Badr, Qiyin Fang, Thomas J. Farrell, Michael J. Farquharson

**Affiliations:** a McMaster University, Department of Physics and Astronomy, Hamilton, Ontario, Canada; b St. Joseph’s Healthcare, Hamilton, Ontario, Canada; c McMaster University, Department of Pathology and Molecular Medicine, Hamilton, Ontario, Canada; d McMaster University, Department of Surgery, Hamilton, Ontario, Canada; e McMaster University, Department of Engineering Physics, Hamilton, Ontario, Canada; f McMaster University, School of Interdisciplinary Science, Hamilton, Ontario, Canada; g Juravinski Hospital and Cancer Center, Hamilton, Ontario, Canada

**Keywords:** fluorescence, diffuse reflectance, optical spectroscopy, breast cancer, breast conservation surgery

## Abstract

**Significance:**

Breast conservation therapy is the preferred technique for treating primary breast cancers. However, breast tumor margins are hard to determine as tumor borders are often ill-defined. As such, there exists a need for a clinically compatible tumor margin detection system.

**Aim:**

A combined time-resolved fluorescence and diffuse reflectance (TRF-DR) system has been developed to determine the optical properties of breast tissue. This study aims to improve tissue classification to aid in surgical decision making.

**Approach:**

Normal and tumor breast tissue were collected from 80 patients with invasive ductal carcinoma and measured in the optical system. Optical parameters were extracted, and the tissue underwent histopathological examination. In total, 761 adipose, 77 fibroglandular, and 347 tumor spectra were analyzed. Principal component analysis and decision tree modeling were performed using only TRF optical parameters, only DR optical parameters, and using the combined datasets.

**Results:**

The classification modeling using TRF data alone resulted in a tumor margin detection sensitivity of 72.3% and specificity of 88.3%. Prediction modeling using DR data alone resulted in greater sensitivity and specificity of 80.4% and 94.0%, respectively. Combining both datasets resulted in the improved sensitivity and specificity of 85.6% and 95.3%, respectively. While both sensitivity and specificity improved with the combined modeling, further study of fibroglandular tissue could result in improved classification.

**Conclusion:**

The combined TRF-DR system showed greater tissue classification capability than either technique alone. Further work studying more fibroglandular tissue and tissue of mixed composition would develop this system for intraoperative use for tumor margin detection.

## 1 Introduction

Breast conservation therapy (BCT) has been the preferable surgical treatment for primary breast cancer for over 30 years.^1^^,^^2^ BCT involves patients undergoing tumor resection to the tumor with a rim of surrounding normal breast tissue and is typically followed by radiation therapy to eradicate residual microscopic disease.^3^ Negative tumor margins are essential in controlling the local recurrence of disease, and the success of BCT is dependent on tumor-free margins.^4^^,^^5^

Conventionally, the gold standard in breast margin assessment is microscopic pathologic examination of the excised tissue.^6^ The tissue is inked to mark orientation, formalin-fixed, paraffin-embedded, hematoxylin and eosin (H&E) stained, and then evaluated by a pathologist. This requires considerable time and labour to be completed. In addition, breast tumor borders are often ill-defined, indistinct, and/or irregularly shaped.^5^ As a result of these challenges, many studies report 7.8% to 50% re-excision rates in patients that underwent BCT.^7^^–^^10^ With no internationally agreed-upon standard margin width set for BCT^11^^,^^12^ and great variability in re-excision rates between surgeons, an intraoperative tool for margin assessment could allow for greater control of disease by providing greater information prior to pathologic examination, reducing positive margins, minimizing the need for reoperation, and morbidity.

Various techniques and commercial devices have been developed for intraoperative margin assessment (IMA), including techniques that utilize electrical properties of tissue, molecular imaging, optical imaging, and optical spectroscopy. Each technique has its own clinical applications and limitations. Techniques using electrical properties of tissue, such as bioimpedance, still suffer from diagnostic capability. MarginProbe^®^ (Dilon Technologies, Newport News, Virginia, United States),^13^ the only commercial device with Food and Drug Administration (FDA) approval for breast IMA, reported sensitivity, specificity, and a false-positive rate in patients of 75.2%, 46.4%, and 53.6%, respectively.^14^ Further studies, notably LeeVan et al. concluded MarginProbe^®^ did not significantly reduce re-excision rates.^15^ Other bioimpedance devices that have not yet been approved for clinical use show promise with greater diagnostic capability, such as ClearEdge™ (LS Biopath, Mountain View, California, United States), where a phase 2 device has shown a sensitivity and specificity of 87.3% and 75.6%, respectively.^16^ However, this device is heavily dependent on a baseline measurement of “normal” breast tissue near the tumor prior to the examination of suspected tissue. This reliance may be an issue if the tissue used for this initial measurement is misidentified. Other IMA devices have been developed that may interrupt existing clinical processes. One prominent commercial device utilizing molecular imaging for breast cancer IMA is the LUM imaging system (Lumicell Inc., Newton, Massachusetts, United States). This device utilizes a novel fluorescent agent, LUM015, which cannot be used in conjunction with isosulfan blue, a dye often used in sentinel node mapping, a common medical procedure to determine whether cancer presence in the body includes the lymph nodes.^17^^,^^18^ The intelligent knife^19^ or iKnife (Medimass, Budapest, Hungary) is a rapid evaporative ionization mass spectrometry tool that has been tested in breast IMA, reporting a sensitivity of 90.9% and specificity of 98.8% in 260 specimens analyzed using their established classification model.^20^ However, this technique is inherently destructive as an electrosurgical unit or an infrared laser is used to thermally ablate tissue resulting in the emission of “surgical smoke.” As such, the interrogated tissue cannot undergo histology for validation.

The only commercial optical imaging device currently approved by the FDA as an investigational device for breast IMA called the OTIS 2.0 (Perimeter Medical Imaging AI Inc., Toronto, Ontario, Canada). A major advantage of this imaging technique is that it is capable of image resolutions on the micron-scale, which is comparable to histological examination.^18^ The pilot study conducted by Munro et al. reported an average image acquisition time of 18.3±5.3  min, with a maximum of 27 min.^21^ This is comparable to conventional frozen section histological examinations, taking approximate 20 to 30 min,^17^ so this device might not reduce the length of a surgical procedure. In addition, it has been reported that the interpretation of optical coherence tomography images suffers from reader variability. A study by Ha et al. found that surgeons reported the lowest sensitivity (76%), specificity (84%), and accuracy (0.82) of all clinicians in the study.^22^ As such, its clinical implementation is disputed.

Raman spectroscopy is an optical spectroscopy technique being investigated for breast cancer IMA. Haka et al. measured *ex vivo* breast specimens and were able to distinguish cancerous from benign tissue with a sensitivity of 83% and specificity of 93%.^23^ A major limitation of Raman spectroscopy is the probability of the interaction, which is significantly lower than other interactions. Raman scatter cross-sections can be 12 to 14 orders of magnitude lower than that of fluorescence.^24^ As a result, fluorescence background must be eliminated from the measured spectra as the fluorescence intensity may be several orders of magnitude greater than Raman signals in biological tissue.^25^ In addition, fluorescence emission occurs over a longer period (nanoseconds) than Raman scattering (picoseconds), adding to the noise that may make Raman spectroscopy challenging.^25^ Although technologies have been and continue to be developed to tackle fluorescence suppression, this often comes with added cost, such as using ultrafast pulsed lasers or increased acquisition time.^25^

Time-resolved fluorescence (TRF) and diffuse reflectance (DR) spectroscopy are optical techniques that can be used to analyze and classify tissue. TRF utilizes a pulse of light to measure the fluorescence of the stimulated tissue over time.^26^ The fluorescence intensity and lifetimes of endogenous fluorophores, such as collagen, nicotinamide adenine dinucleotide (NADH), and flavin adenine dinucleotide (FAD), can be extracted and used in tissue analysis.^27^ DR spectroscopy utilizes a broadband light source to stimulate tissue, and various detection fibers arranged in different geometries can be used to observe the reflectance of the light.^28^ The reflectance can be used to extract the absorption and reduced scattering coefficients at measurement wavelengths to analyze tissue.^27^

Various TRF studies have found differences in the fluorescence intensities and lifetimes of collagen, NADH, and FAD between cancerous and normal breast tissue.^29^^–^^33^ Similarly, various DR studies have utilized the spectral reflectance, absorption coefficient, and reduced scattering coefficient to analyze breast tissue.^34^^–^^39^ Several studies have tested the capabilities of combined fluorescence and reflectance systems for breast tissue classification, with sensitivities ranging from 70% to 100% and specificities ranging from 75% to 96%;^40^^–^^43^ however, each study involved samples collected from a maximum of 45 cancer patients.

The biological emission and reflectance (BEAR) system combines TRF-DR to investigate the classification capabilities of TRF alone, DR alone, and TRF-DR combined. The goal of this work is to investigate the possibility of improving tissue classification modeling using multiple optical techniques and collecting a greater number of spectra from a greater number of patients. The development of a technique that allows for rapid margin assessment can improve confidence in the operating room until pathological assessment can be performed. If successful, this non-destructive investigation technique and system could complement existing clinical processes and reduce both the time required for surgery and re-excision rates.

## 2 Materials and Methods

### 2.1 Samples and Histological Data

Normal and tumor breast samples were collected from 80 patients with invasive ductal carcinoma following lumpectomy from St. Joseph’s Hospital, Hamilton, Ontario, Canada [Hamilton Integrated Research Ethics Board (Project No. 10-3393)]. Tumor samples were collected near the center of the tumor; whereas, normal samples were collected from the exterior of the excised tissue. Samples were frozen and stored at $-80^{{}^{\circ}}C$. A study by Shalaby et al.) showed that freezing and thawing normal and tumor breast tissue did not result in significant differences in optical properties.^27^ Prior to measurement, samples were thawed and trimmed to fill a sample holder with a 12.7 mm diameter and 1 mm deep cylindrical sample holder. The holders are covered on both sides using optically transparent ultralene film to hold the samples in place and flatten the tissue surface.

After measurement, samples were prepared for histological analysis. Samples were fixed in formalin, alcohol washed, and embedded in paraffin to form tissue blocks. Tissue blocks were sectioned and mounted onto glass slides for H&E staining. Following staining, the glass slides were digitized and registered with optical raster scans to quantify 1 mm × 1 mm pixel divisions. Each pixel was evaluated by a pathologist to classify the tissue composition as percent tumor, fibroglandular, and/or adipose.

### 2.2 Instrumentation

**Fig. 1:**
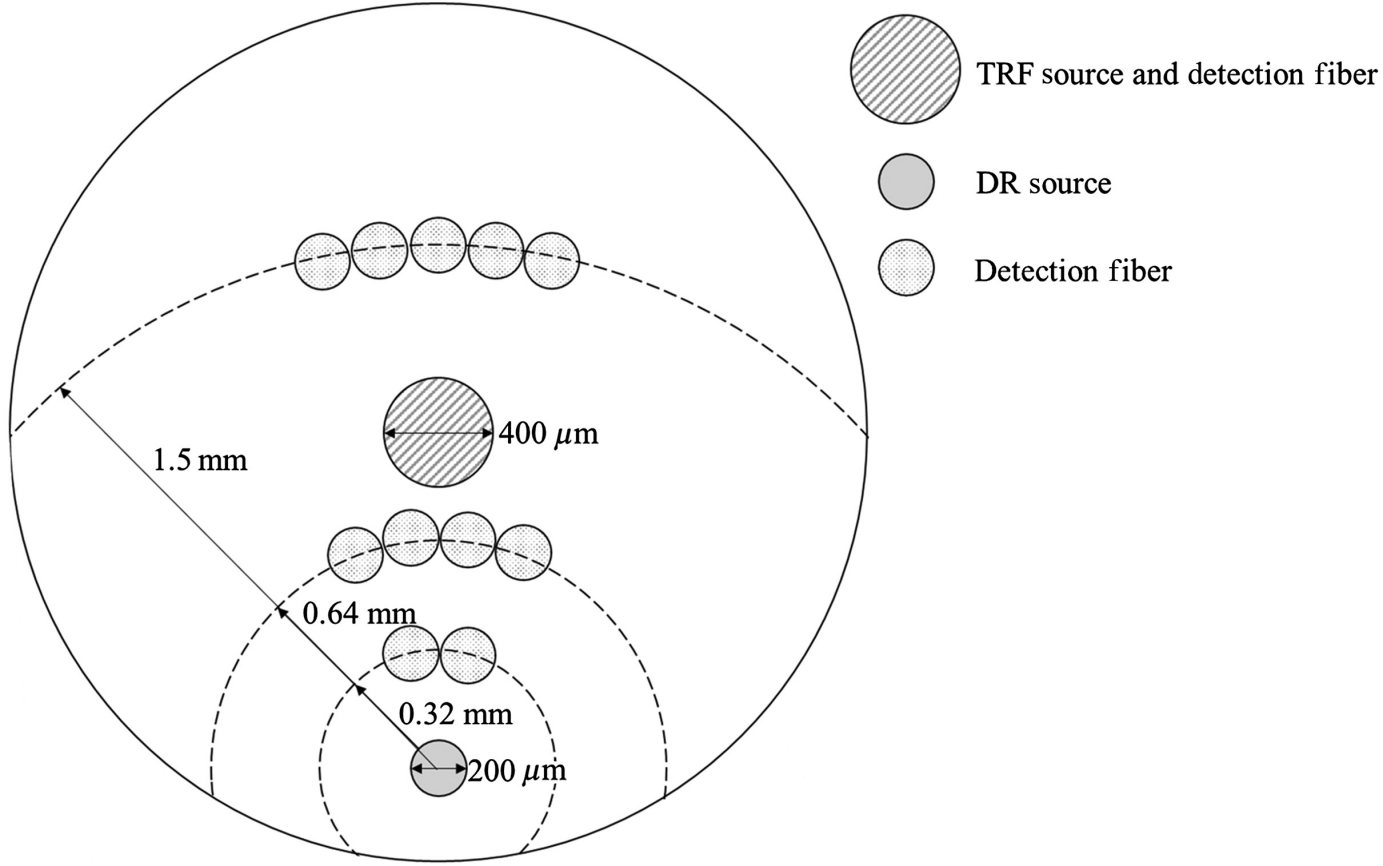
Geometry of the 3 mm diameter measurement probe containing a 400  μm diameter TRF excitation and collection fiber, a 200  μm DR excitation fiber, and three detection fiber bundles, 0.32 mm, 0.64 mm, and 1.5 mm from the DR excitation fiber.

Mounted samples were placed in the BEAR system for measurement.^44^ This combined system uses one 3 mm diameter custom probe, as shown in Fig. 1, to make spot measurements. The probe was positioned ∼1  mm from the surface of the tissue for each sample measured. At the center of the probe is a 400  μm diameter fiber used for TRF excitation using a 355 nm Nd:YAG laser (PNV-M02510-130, Teem Photonics, France) and detection. An acousto-optical tunable filter (TEAF5-0.36-0.52-S-MSD, Brimrose, United States) is used to measure fluorescence wavelengths from 380 to 570 nm in increments of 5 nm. The laser is triggered to send 11 pulses per wavelength with a power of ∼3  mW at the tissue surface. Fluorescence is measured using a photomultiplier tube (C5594-12, Hamamatsu Photonics, Hamamatsu City, Japan) biased to 2000 V. The output was then sampled using a digitizer (ADQ7DC, 14 bit, 10 GS/s, 2.5 GHz bandwidth, SP Devices, Linköping, Sweden). The laser is pulsed to make 11 measurements per spot.

The probe also contains a 200  μm diameter fiber used for DR excitation using a broadband Xenon lamp (ASB-XE-175BF, Spectral Products, Putnam, Connecticut, United States) as well as multiple 200  μm diameter fibers used for reflectance detection. These DR detection fibers are bundled such that there are three detection bundles 0.32 mm, 0.64 mm, and 1.5 mm from the DR excitation source fiber. Each bundle is connected to a separate spectrometer (BW-UVIS, 600 g/mm, 50  μm slit, StellarNet, United States) to record reflectance.

**Fig. 2:**
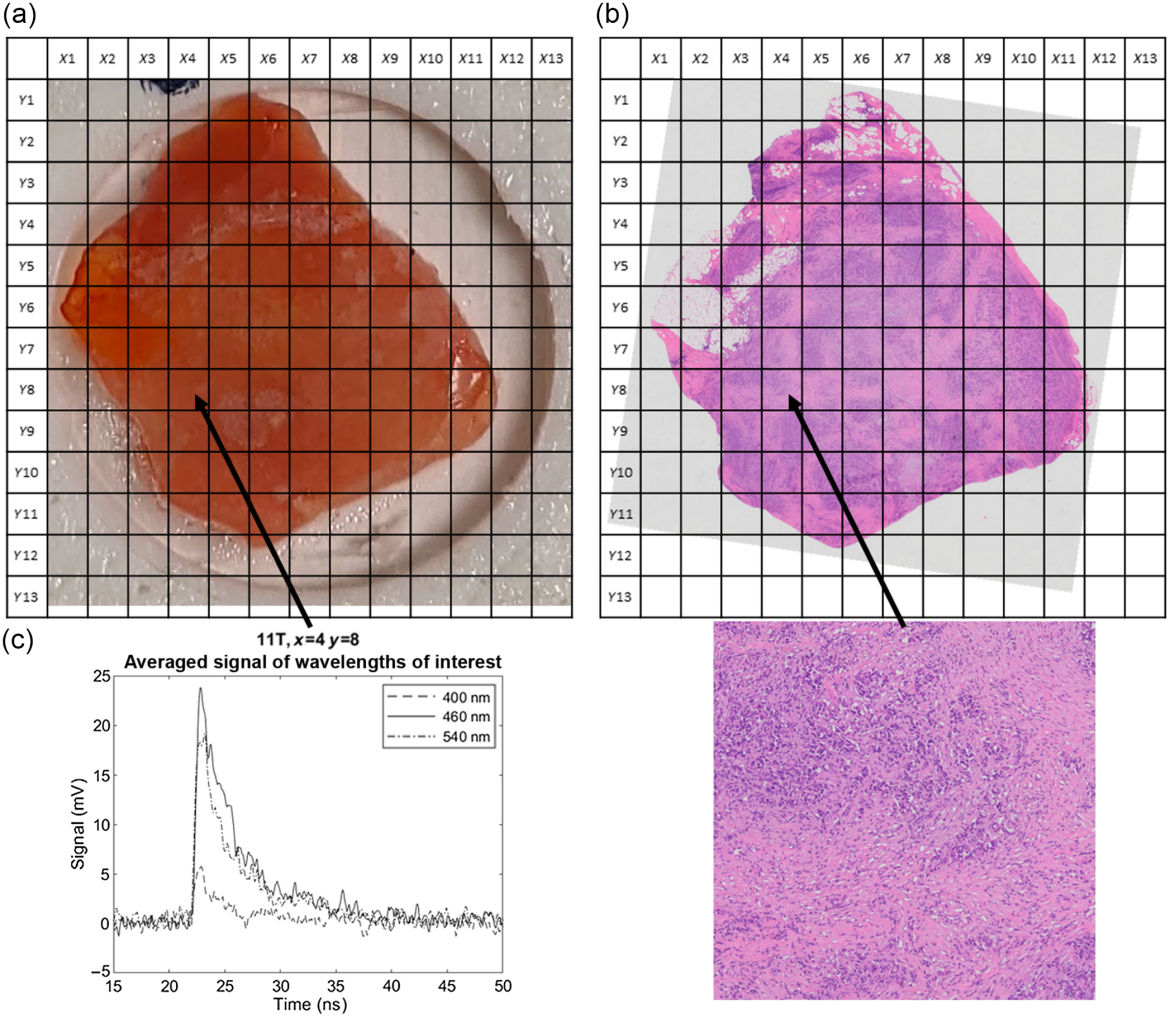
Photograph of the sample (a) and histology slide (b) of sample 11T. Grid overlays depict raster scan locations of each spot measurement. The averaged signal for some wavelengths of interest (400 nm, 460 nm, and 540 nm) for spot x=4, y=8 (c) and its corresponding histology image used for tissue classification (d) are also shown.

Samples were placed on an x-y linear stage (XLS-1-80-1250, Xeryon, Leuven, Belgium) to translate the samples for raster scanning. Samples were translated in 1  mm×1  mm step sizes and a spot measurement was performed at each location. Figure 2 shows a photograph of a sample and its corresponding histology slide with grids overlaid depicting the raster scan. An averaged spectrum for one of the spot measurements and the corresponding region in the histology image is shown below. Raster scanning enabled rapid measurement of the entire sample surface, allowing for a greater number of spectra to be collected in a shorter time period. All components were connected to a computer to automatically control the timing and execution of sample excitation, measurement, stage translation, and recording.

Each spot measurement takes approximately 1 s. The measurement time increases during raster scanning as spectra from each spot need to be saved and the translation stage requires time to move to the next position. In addition, the acquisition software performs coarse analysis to generate live-time data during the scan. As such, the time for each spot measurement increases to approximately 5 s during raster scanning. The speed of the imaging is mostly dependent on the step size of the raster scan. For a 13  mm×13  mm area raster scanned with 1  mm×1  mm step sizes, the measurement time is approximately 15 min.

### 2.3 Data Processing and Analysis

Following measurement, the measured signal at each wavelength was averaged for the 11 laser pulses. Custom MATLAB (v 9.12; MathWorks, Natick, Massachusetts, United States) code was developed to fit the average spectra to extract the fluorescence intensity and lifetime of biomolecules of interest. The biomolecules of interest and their emission wavelengths are reported in Table 1. Fluorescence intensities of all emission wavelengths for each biomolecule were averaged and then normalized to the NADH intensity at 460 nm, the mean emission wavelength of NADH for each pixel. As such, the reported fluorescence intensities are collagen/NADH and FAD/NADH ratios. This normalization accounts for slight variations in sample to detector distance between measurements. In total, five TRF parameters were quantified: collagen/NADH intensity, FAD/NADH intensity, collagen lifetime, NADH lifetime, and FAD lifetime.

**Table 1:** Biomolecules of interest and emission wavelengths with 355 nm excitation.

Biomolecule	Emission wavelengths (355 nm excitation)
Collagen	380 to 440 nm^33^^,^^45^^,^^46^
NADH	450 to 500 nm^45^^,^^46^
FAD	520 to 570 nm^45^^–^^47^

A sample filled with de-ionized water was measured as background and subtracted from reflectance measurements collected from the second DR detection fiber bundle (0.64 mm). The reflectance data were then normalized to a 99% DR standard (Labsphere Inc., North Sutton, New Hampshire, United States). Optical phantoms made of India Ink (Higgins Ink, Leeds, Massachusetts United States) and 1  μm-diameter polystyrene microspheres (Polysciences, Warrington, Pennsylvania, United States) were measured, and the reflectance data were used to create a look-up table for the extraction of the absorption coefficient, $\mu_{a}$, and reduced scattering coefficient, $\mu_{s}^{\prime }$, following the methods outlined by Le et al.^46^ This look-up table allowed for the $\mu_{a}$ and $\mu_{s}^{\prime }$ at each wavelength to be extracted using the measured reflectance of each measurement. In total, eight DR parameters were quantified: normalized reflectance at 520 nm, normalized reflectance at 560 nm, $\mu_{a}$ at 540 nm, $\mu_{a}$ at 560 nm, $\mu_{a}$ at 576 nm, $\mu_{s}^{\prime }$ at 540 nm, $\mu_{s}^{\prime }$ at 560 nm, and $\mu_{s}^{\prime }$ at 576 nm. Greater detail regarding the selection of these parameters is outlined in Dao et al.^44^

Multivariate analysis was performed using Orange software (v 3.32)^48^ using extracted optical parameters from pixels classified as 100% tumor, fibroglandular, or adipose. In total, 761 adipose, 77 fibroglandular, and 347 tumor spectra were analyzed from 80 patients. Principal component analysis (PCA) was performed on three datasets: TRF parameters only, DR parameters only, and combined TRF and DR parameters. The number of principal components (PCs) was increased until it covered at least 98% of the variance in each dataset. There were four PCs used for the TRF and DR datasets and eight PCs in the combined dataset. The PCA loadings are included in the Supplementary Material. Decision tree modeling was also performed using Orange software. Classification was non-binary. The decision tree model classified tissue as adipose, fibroglandular, or adipose. The classifier was set to include a minimum number of three instances in leaves, not to split subsets smaller than five, and to stop when the majority reaches 95%. Tissue classification was performed using “leave-one-patient-out” cross-validation where the data from all but one patient is modeled and the data from one patient is tested and scored using this model. This process is then repeated for all patients in this study. Following testing, the receiver operating characteristic (ROC) curve was generated using the predictions from all folds.

To determine whether modeling with combined optical parameters improved tumor classification compared to either technique alone, McNemar-Bowker tests were conducted. The classification of the tumor pixels were compared between modeling using just TRF parameters and modeling using the combined TRF-DR parameters. Similarly, a second test was conducted to compare classification of tumor pixels using just DR parameters and the combined TRF-DR parameters.

## 3 Experimental Results and Discussion

### 3.1 Time-Resolved Fluorescence Only

**Fig. 3:**
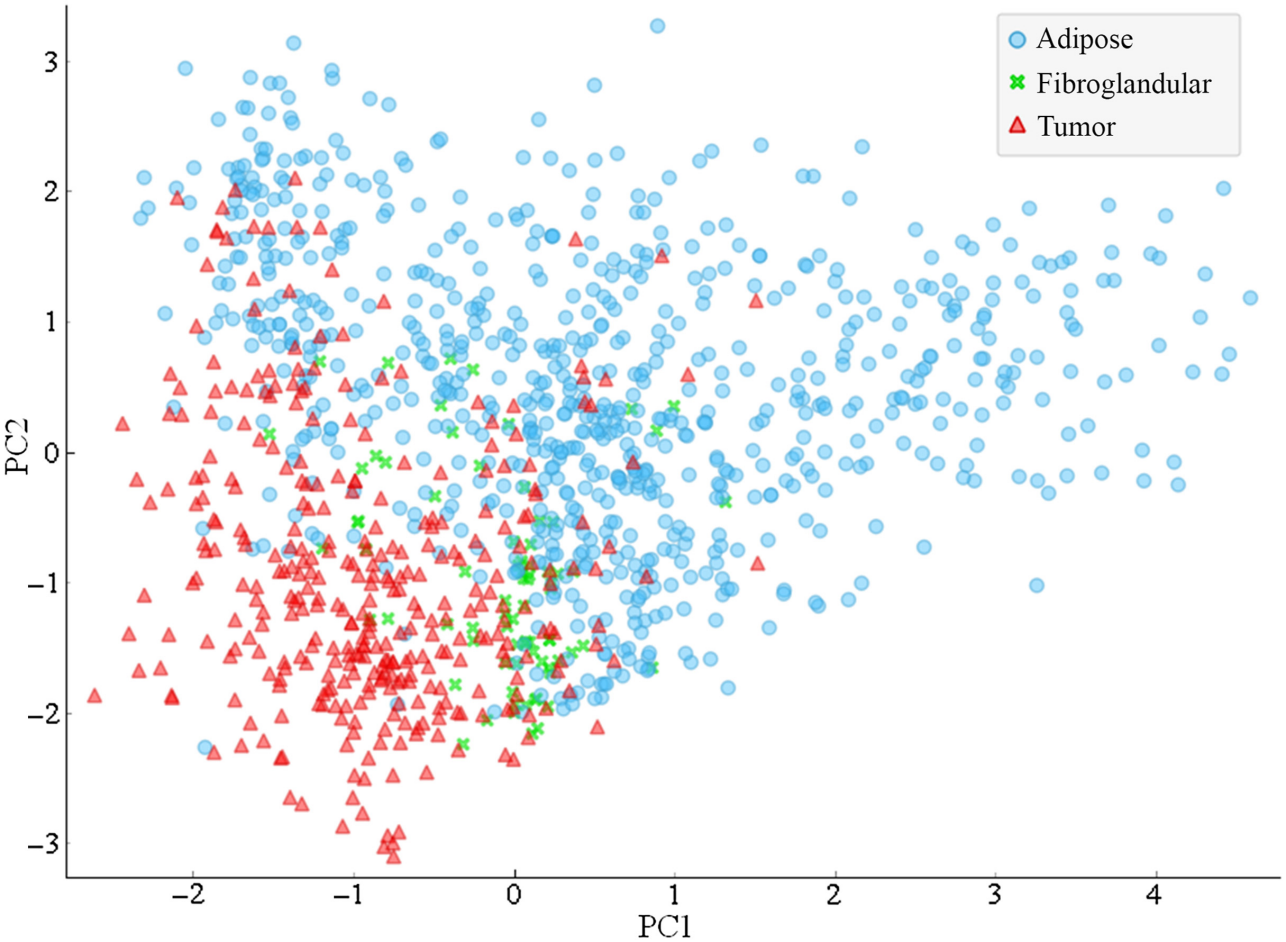
Results of PCA on TRF dataset. PC1 and PC2 are plotted for adipose, fibroglandular, and tumor tissue.

The fluorescence intensities of collagen and FAD as well as the fluorescence lifetimes of collagen, NADH, and FAD were used to create the TRF dataset. Four components were used to cover 99.2% of the variance in the TRF dataset. The two PCs covering the greatest variance, PC1 covering 43% of the variance and PC2 covering 34% of the variance, are plotted in Fig. 3. While the PCA showed some separation between tumor and adipose tissue in PC1, there is significant overlap between fibroglandular and the other tissue types. PC2 did not show much separation between tissue types.

**Fig. 4:**
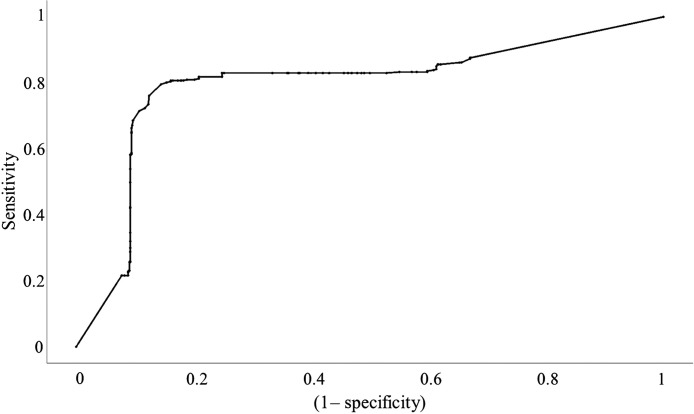
ROC for tumor classification using TRF dataset.

Tissue classification was performed using decision tree modeling with only the TRF parameters. The results are shown in Table 2. The ROC curve for tumor classification is displayed in Fig. 4. The sensitivity, specificity, and area under the ROC curve (AUC) of classifying each tissue group are reported in Table 3.

**Table 2:** Tissue classification using decision tree modeling with TRF parameters only.

		Predicted		
		Adipose	Fibroglandular	Tumor
Actual	Adipose	691	15	55
	Fibroglandular	23	11	43
	Tumor	72	24	251

**Table 3:** Sensitivity, specificity, and AUC of classification of adipose, fibroglandular, and tumor samples using TRF parameters.

	Adipose	Fibroglandular	Tumor
Sensitivity	90.8%	14.3%	72.3%
Specificity	77.6%	96.5%	88.3%
AUC	0.882	0.664	0.842

Modeling using only TRF parameters proved successful for adipose with a sensitivity of 90.8%, specificity of 77.6%, and AUC of 0.882. Tumor classification had a high specificity of 88.3%, but lower sensitivity and AUC of 72.3% and 0.842, respectively. TRF parameters did not classify fibroglandular tissue well, with a very low sensitivity of 14.3%. Therefore, the classification ability of TRF alone would allow for tissue classification in general but would not provide greater insight on the normal tissue.

### 3.2 Diffuse Reflectance

**Fig. 5:**
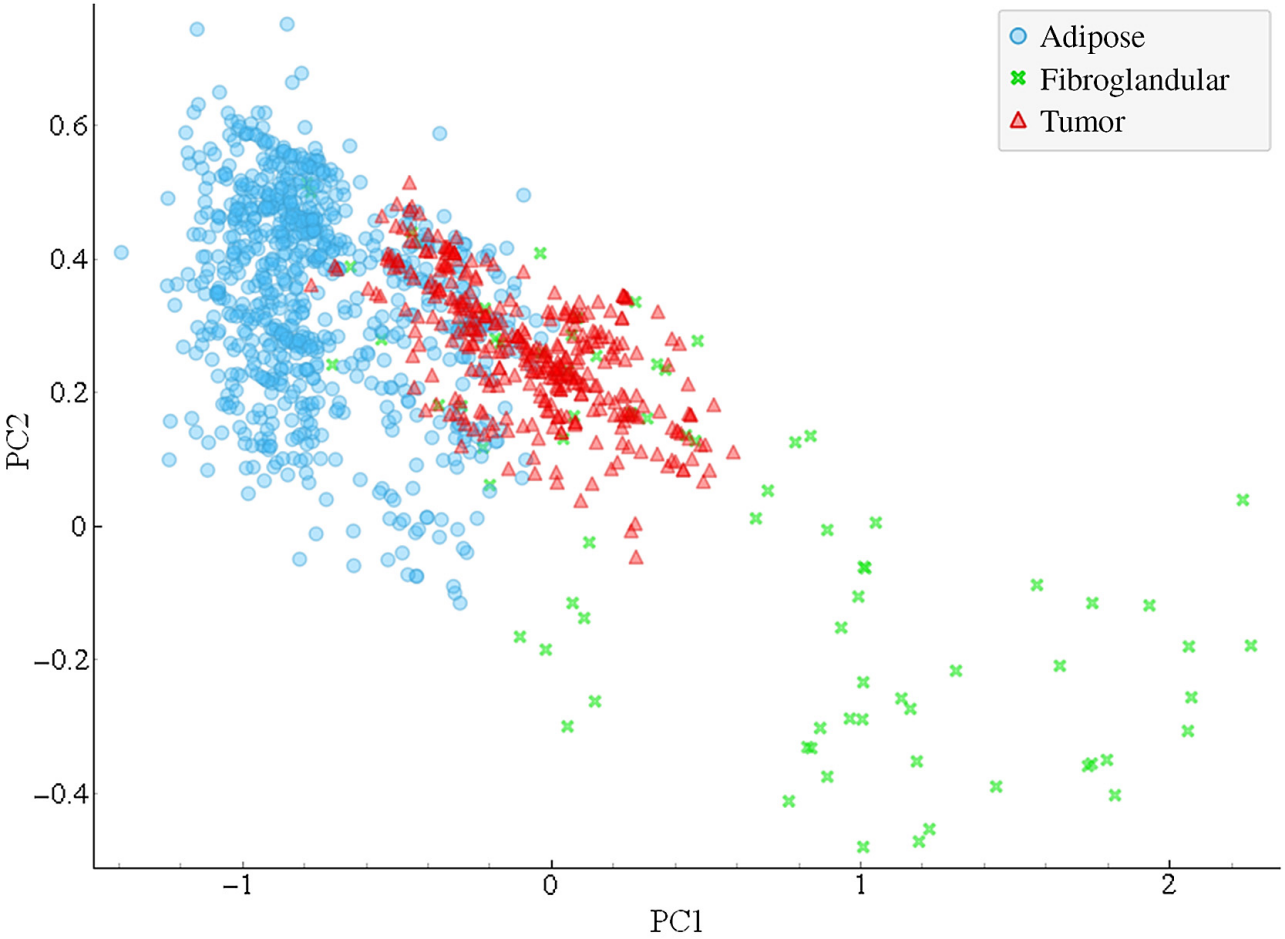
Results of PCA on DR dataset. PC1 and PC2 are plotted for adipose, fibroglandular, and tumor tissue.

The normalized reflectance at 520 nm and 560 nm as well as the $\mu_{a}$ and $\mu_{s}^{\prime }$, at 540, 560, and 576 nm were used to create the DR dataset. Four components were used to cover 98.5% of the variance in the DR dataset. The two PCs covering the greatest variance, PC1 covering 59% of the variance and PC2 covering 32% of the variance, are plotted in Fig. 5. The PCA using DR parameters showed greater clustering of adipose and tumor samples; however, the fibroglandular tissue showed greater variability within the group. PC1 showed greater separation of tissue groups than PC2, with PC 2 showing very little difference between tumor and adipose.

**Fig. 6:**
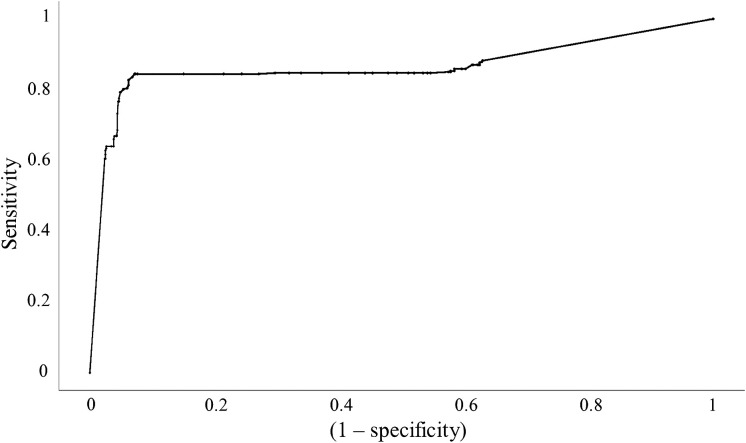
ROC for tumor classification using DR dataset.

Tissue classification was performed using decision tree modeling with only the DR parameters. The results are shown in Table 4. Figure 6 shows the ROC curve for tumor classification. The sensitivity, specificity, and AUC of classifying each tissue group are reported in Table 5. Modeling using DR parameters greatly improved the classification ability for all tissue types when compared to TRF modeling. The greatest improvement was the fibroglandular sensitivity increasing from 14.3% to 63.6%. Better classification of fibroglandular tissue resulted in significantly fewer incorrect classifications of tumor, improving its sensitivity as well. As such, DR parameters are shown to be more effective at tissue classification than TRF parameters.

**Table 4:** Tissue classification using decision tree modeling with DR parameters only.

		Predicted		
		Adipose	Fibroglandular	Tumor
Actual	Adipose	729	2	30
	Fibroglandular	8	49	20
	Tumor	60	8	279

**Table 5:** Sensitivity, specificity, and AUC of classification of adipose, fibroglandular, and tumor samples using DR parameters.

	Adipose	Fibroglandular	Tumor
Sensitivity	95.8%	63.6%	80.4%
Specificity	84.0%	99.1%	94.0%
AUC	0.905	0.883	0.887

### 3.3 Dual Time-Resolved Fluorescence-Diffuse Reflectance

**Fig. 7:**
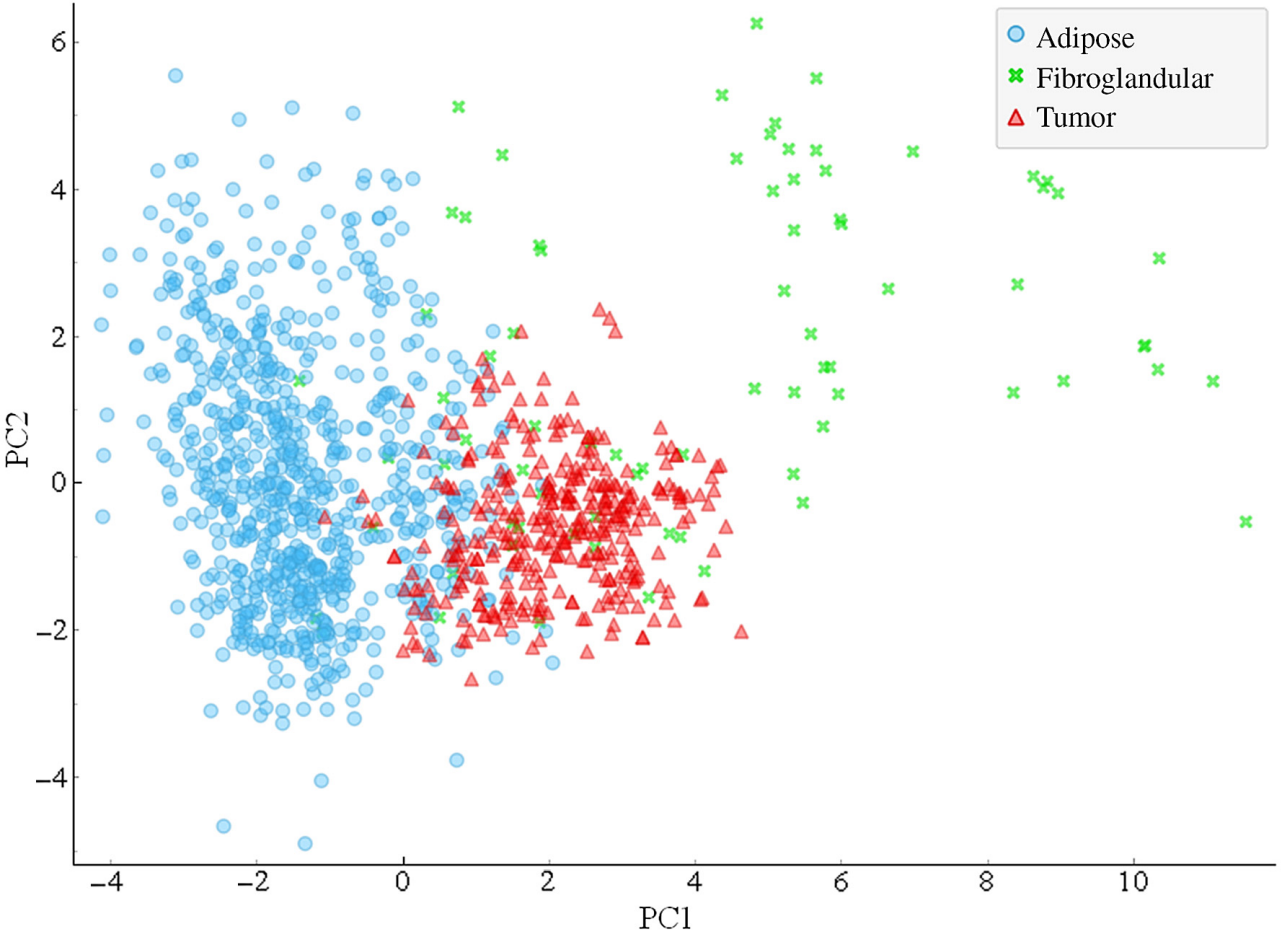
Results of PCA on combined TRF-DR dataset. PC-1 and PC-2 are plotted for adipose, fibroglandular, and tumor tissue.

The TRF and DR datasets were combined to form the dual TRF-DR dataset. Eight components were used to cover 98.9% of the variance in the combined dataset. The two PCs covering the greatest variance, PC1 covering 42% of the variance and PC2 covering 21% of the variance, are plotted in Fig. 7. The PCA using both TRF and DR parameters showed similar clustering of tissue groups, with most of the separation resulting from PC1.

**Fig. 8:**
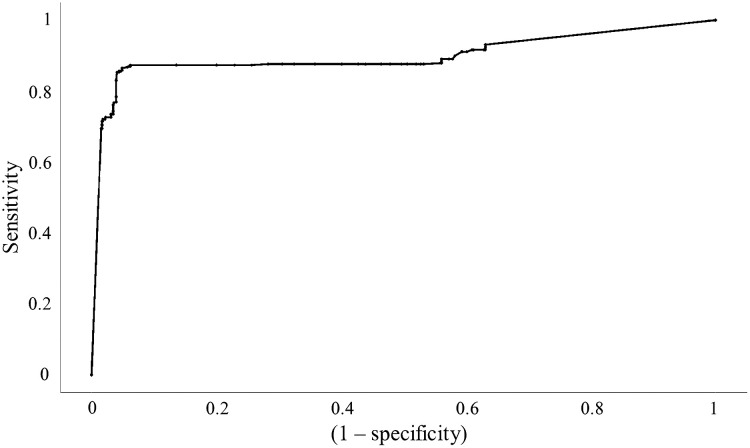
ROC for tumor classification using the combined TRF-DR dataset.

Tissue classification was performed using decision tree modeling with both TRF and DR parameters. The results are shown in Table 6. The ROC curve for the combined dataset is shown in Fig. 8. The sensitivity, specificity, and AUC of classifying each tissue group are reported in Table 7. Modeling using both TRF and DR parameters further improved the classification ability for all tissue types.

**Table 6:** Tissue classification using decision tree modeling with combined TRF-DR parameters.

		Predicted		
		Adipose	Fibroglandular	Tumor
Actual	Adipose	736	3	22
	Fibroglandular	11	49	17
	Tumor	44	6	297

**Table 7:** Sensitivity and specificity of classification of adipose, fibroglandular, and tumor samples using combined TRF and DR parameters.

	Adipose	Fibroglandular	Tumor
Sensitivity	96.7%	63.6%	85.6%
Specificity	87.0%	99.2%	95.3%
AUC	0.923	0.876	0.911

Combining both optical techniques improves tissue classification compared to either technique alone, as shown in Table 8, summarizing the sensitivity, specificity, and AUC using each technique. Combining datasets did not improve the sensitivity of fibroglandular classification, but it did reduce the misclassification of some fibroglandular measurements as tumor from 20 false positives using the DR dataset only to 17 using the combined dataset.

**Table 8:** Summary of sensitivity, specificity, and AUC of positive margin (tumor) classification using each dataset.

	TRF only	DR only	Combined TRF and DR
Sensitivity	75.2%	82.7%	83.9%
Specificity	90.8%	95.3%	96.2%
AUC	0.842	0.887	0.911

For each tumor pixel in this study (n=347), the classification for each model was examined to determine whether combining TRF and DR parameters improved tumor classification. Table 9 shows tumor classification using only TRF parameters and how the classifications changed following modeling using the combined parameters compare. In 50 instances, tumor pixels that were incorrectly classified as adipose using modeling with only TRF parameters were then correctly classified as tumor using the combined dataset. Similarly, 23 of the tumor pixels incorrectly classified as fibroglandular using TRF parameters were then correctly classified using the combined parameters. However, for 27 tumor pixels correctly classified using only TRF parameters, 22 were mis-classified as adipose and 5 as fibroglandular using the combined dataset. Overall, combining the parameters resulted in 46 more correctly classified pixels, a 13.3% increase. With a McNemar–Bowker test p-value of <0.01, there is a statistically significant difference between the two classification models.

**Table 9:** Changes to tumor classification using TRF parameters only and combined TRF and DR parameters.

		Tumor classification using combined TRF and DR parameters		
		Adipose	Fibroglandular	Tumor
Tumor classification using TRF parameters only	Adipose	22	0	50
	Fibroglandular	0	1	23
	Tumor	22	5	224

Similarly, Table 10 shows the changes to tumor classification using only DR parameters and the combined TRF and DR parameters. In this comparison, 35 more tumor pixels were correctly classified and 17 more were mis-classified using the combined dataset than just DR parameters, resulting in an increase of 18 more correct classifications, an increase of 5%. With a McNemar–Bowker test p-value of 0.02, there is a statistically significant difference between the two classification models. Both comparisons show that combining the parameters from both techniques results in improved tumor classification compared to either technique alone.

**Table 10:** Changes to tumor classification using TRF parameters only and combined TRF and DR parameters.

		Tumor classification using combined TRF and DR parameters		
		Adipose	Fibroglandular	Tumor
Tumor classification using DR parameters only	Adipose	28	0	32
	Fibroglandular	1	4	3
	Tumor	15	2	262

During sample collection, obtaining normal adipose tissue is much easier than obtaining normal fibroglandular tissue. As such, sampling of normal breast issue was biased toward adipose. This resulted in the sample size for adipose to be greater than fibroglandular by nearly 10-fold. In future work, it will be important to collect more fibroglandular tissue for better classification and to decrease the number of false positives.

While this technology does not yet have a tumor detection sensitivity and specificity high enough to replace microscopic pathologic examination as the gold standard in breast margin assessment, it does provide information that could give surgeons greater confidence during surgery. The system could aid in real-time decision making in the operating room, reducing surgery time, which can improve patient outcome.

## 4 Conclusion

In this work, it was shown that combining TRF and DR measurements for tissue classification is more beneficial than using either technique individually. While discrimination of adipose and tumor tissue is quite successful, the extent to which tumor margins can be detected is heavily dependent on the ability to classify fibroglandular tissue. Therefore, emphasis is placed on the need for greater study and sampling of normal fibroglandular tissue. With shown improvements in both sensitivity and specificity, the BEAR system shows potential as a surgical tool for intraoperative breast tumor margin detection, complementing existing microscopic pathological examination. Future research will involve modeling spectra from tissue of mixed composition and testing of fresh specimens in the operating room to confirm the feasibility and suitability of the BEAR system in a clinical environment. The eigenvectors of the PCs will also be examined to determine which optical parameters are optimal for tissue classification. In addition, investigation into the use of software for histology classification will be explored, which could reduce the burden of analysis from pathologists, improving clinical implementation.

## Code, Data, and Materials Availability

The archived version of the supporting data and code can be freely accessed and executed through Code Ocean.
