# Supplementary material for: Multivariate analysis of breast tissue using optical parameters extracted from a combined time-resolved fluorescence and diffuse reflectance system for tumor margin detection
Source: J Biomed Opt. 2023 Aug 23;28(8):085001. doi: 10.1117/1.JBO.28.8.085001 (PMC10445658; doi:10.1117/1.JBO.28.8.085001)
Supplement: Supplementary file 1 [file JBO_028_085001_SD001.pdf]

Supplementary Table 1: PCA Loadings for TRF Parameters Only

Component	Variance	Collagen Normalized Intensity	FAD Normalized Intensity	Collagen Lifetime	NADH Lifetime	FAD Lifetime
PC1	0.429591	-0.470906	0.352066	0.262179	0.474497	0.600343
PC2	0.341431	-0.283856	0.590125	0.422695	-0.52956	-0.334774
PC3	0.166309	0.59185	-0.103704	0.782306	0.151252	0.0638685
PC4	0.054791	0.588258	0.68461	-0.374449	0.0148548	0.211729

Supplementary Table 2: PCA Loadings for DRS Parameters Only

Component	Variance	Reflectance at 520nm	Reflectance at 560nm	μ_a at 540nm	μ_a at 560nm	μ_a at 576nm	μ_s' at 540nm	μ_s' at 560nm	μ_s' at 576nm
PC1	0.593058	0.439834	0.4304	0.115998	0.0722039	0.120767	0.441194	0.448299	0.438663
PC2	0.322643	0.0515349	0.103447	-0.59709	-0.536382	-0.567329	0.0720533	0.0816827	0.0932523
PC3	0.0440848	0.0881875	0.0502263	0.210874	-0.807102	0.528939	-0.076785	-0.0661332	-0.061423
PC4	0.0253298	0.489732	0.601158	-0.0323352	0.119102	-0.0795711	-0.313043	-0.366084	-0.381046

Supplementary Table 3: PCA Loadings for Combined TRF and DR Parameters

PC	Variance	Collagen Normalized Intensity	FAD Normalized Intensity	Collagen Lifetime	NADH Lifetime	FAD Lifetime	Reflectance at 520nm	Reflectance at 560nm	μ_a at 540nm	μ_a at 560nm	μ_a at 576nm	μ_s' at 540nm	μ_s' at 560nm	μ_s' at 576nm
PC1	0.419629	0.238316	-0.266903	-0.15749	-0.02302	-0.13378	0.407373	0.384995	0.128632	0.085006	0.132292	0.398072	0.402267	0.391165
PC2	0.213499	-0.111951	0.0109913	-0.01227	0.284307	0.285985	0.088404	0.15503	-0.52283	-0.47636	-0.49457	0.113982	0.125895	0.137906
PC3	0.135003	-0.138382	-0.120217	-0.03258	0.642777	0.603661	-0.00791	0.028935	0.266416	0.220384	0.259383	-0.00569	-0.00283	-0.00131
PC4	0.09499	-0.377218	0.586312	0.585025	-0.10058	0.05718	0.076442	0.152213	0.091638	0.084653	0.086301	0.171512	0.190972	0.200983
PC5	0.06308	0.655397	-0.17938	0.699686	0.143821	0.043153	0.003122	-0.04379	-0.01491	-0.12566	0.022853	-0.04337	-0.04631	-0.06068
PC6	0.026604	-0.147748	-0.0356746	-0.02712	-0.0259	-0.04447	0.076174	0.037873	0.211074	-0.79811	0.526225	-0.06319	-0.04913	-0.03769
PC7	0.021262	0.548363	0.689605	-0.36291	0.012591	0.198743	0.003366	0.189146	0.035892	-0.06075	0.064766	-0.04837	-0.04662	-0.05396
PC8	0.014994	-0.126244	-0.0583869	0.090599	-0.0026	-0.05766	0.489566	0.601156	-0.02828	0.110112	-0.07155	-0.31098	-0.35434	-0.35741
